# Twelve-Month Follow-up of the Immune Response After COVID-19 Vaccination in Patients with Genitourinary Cancers: A Prospective Cohort Analysis

**DOI:** 10.1093/oncolo/oyad067

**Published:** 2023-03-27

**Authors:** Luis Meza, Zeynep Zengin, Sabrina Salgia, Jasnoor Malhotra, Ewa Karczewska, Tanya Dorff, Abhishek Tripathi, Jennifer Ely, Erin Kelley, Heather Mead, JoAnn Hsu, Nazli Dizman, Nicholas Salgia, Neal Chawla, Alex Chehrazi-Raffle, Ramya Muddasani, Ameish Govindarajan, Adam Rock, Sandy Liu, Ravi Salgia, Jeffrey Trent, John Altin, Sumanta K Pal

**Affiliations:** Department of Medical Oncology and Experimental Therapeutics, City of Hope Comprehensive Cancer Center, Duarte, CA, USA; Department of Medical Oncology and Experimental Therapeutics, City of Hope Comprehensive Cancer Center, Duarte, CA, USA; Department of Medical Oncology and Experimental Therapeutics, City of Hope Comprehensive Cancer Center, Duarte, CA, USA; Department of Medical Oncology and Experimental Therapeutics, City of Hope Comprehensive Cancer Center, Duarte, CA, USA; Department of Immuno-Oncology, City of Hope Comprehensive Cancer Center, Duarte, CA, USA; Department of Medical Oncology and Experimental Therapeutics, City of Hope Comprehensive Cancer Center, Duarte, CA, USA; Department of Medical Oncology and Experimental Therapeutics, City of Hope Comprehensive Cancer Center, Duarte, CA, USA; Pathogen and Microbiome Division, Translational Genomics Research Institute North, Flagstaff, AZ, USA; Pathogen and Microbiome Division, Translational Genomics Research Institute North, Flagstaff, AZ, USA; Pathogen and Microbiome Division, Translational Genomics Research Institute North, Flagstaff, AZ, USA; Department of Medical Oncology and Experimental Therapeutics, City of Hope Comprehensive Cancer Center, Duarte, CA, USA; Department of Medical Oncology and Experimental Therapeutics, City of Hope Comprehensive Cancer Center, Duarte, CA, USA; Department of Internal Medicine, Yale University School of Medicine, New Haven, CT, USA; Department of Medical Oncology and Experimental Therapeutics, City of Hope Comprehensive Cancer Center, Duarte, CA, USA; Department of Medical Oncology and Experimental Therapeutics, City of Hope Comprehensive Cancer Center, Duarte, CA, USA; Department of Medical Oncology and Experimental Therapeutics, City of Hope Comprehensive Cancer Center, Duarte, CA, USA; Department of Medical Oncology and Experimental Therapeutics, City of Hope Comprehensive Cancer Center, Duarte, CA, USA; Department of Medical Oncology and Experimental Therapeutics, City of Hope Comprehensive Cancer Center, Duarte, CA, USA; Department of Medical Oncology and Experimental Therapeutics, City of Hope Comprehensive Cancer Center, Duarte, CA, USA; Department of Medical Oncology and Experimental Therapeutics, City of Hope Comprehensive Cancer Center, Duarte, CA, USA; Department of Medical Oncology and Experimental Therapeutics, City of Hope Comprehensive Cancer Center, Duarte, CA, USA; Integrated Cancer Genomics Division, Translational Genomics Institute, Phoenix, AZ, USA; Pathogen and Microbiome Division, Translational Genomics Research Institute North, Flagstaff, AZ, USA; Department of Medical Oncology and Experimental Therapeutics, City of Hope Comprehensive Cancer Center, Duarte, CA, USA

**Keywords:** COVID-19 vaccine, genitourinary cancer, immune response

## Abstract

**Background:**

Vaccinations against severe acute respiratory syndrome coronavirus 2 (SARS-CoV-2) have had a transformative impact on morbidity and mortality. However, the long-term impact of vaccination on patients with genitourinary cancers is currently unknown.

**Materials and Methods:**

This study aimed to assess seroconversion rates in patients with genitourinary cancers receiving COVID-19 vaccination. Patients with prostate cancer, renal cell carcinoma, or urothelial cancer who had not been vaccinated for COVID-19 were included. Blood samples were obtained at baseline and after 2, 6, and 12 months of one dose of an FDA-approved COVID-19 vaccine. Antibody titer analysis was performed using the SCoV-2 Detect IgG ELISA assay, and the results were reported as immune status ratio (ISR). A paired *t*-test was used for comparison of ISR values between timepoints. In addition, T-cell receptor (TCR) sequencing was performed to assess for differences in TCR repertoire 2 months after vaccination.

**Results:**

Out of 133 patients enrolled, 98 baseline blood samples were collected. At 2-, 6-, and 12-month time points 98, 70, and 50 samples were collected, respectively. Median age was 67 (IQR, 62-75), with the majority of patients diagnosed with prostate (55.1%) or renal cell carcinoma (41.8%). Compared to baseline (0.24 [95% CI, 0.19-0.31]) a significant increase in the geometric mean ISR values was observed at the 2-month timepoint (5.59 [4.76-6.55]) (*P* < .001). However, at the 6-month timepoint, a significant decrease in the ISR values was observed (4.66 [95% CI, 4.04-5.38]; *P* < .0001). Notably, at the 12-month timepoint, the addition of a booster dose resulted in an absolute increase in the ISR values compared to those who did not receive a booster dose (*P* = .04).

**Conclusions:**

Only a minority of patients with genitourinary cancers did not ultimately achieve satisfactory seroconversion after receiving commercial COVID-19 vaccination. Cancer type or treatment rendered did not appear to affect the immune response mounted after vaccination.

## Teaser

The long-term effect of vaccination against SARS-CoV-2 on patients with genitourinary cancers is currently unknown. This study assessed the long-term effect of commercially available COVID-19 vaccination in patients with renal cell carcinoma, urothelial carcinoma, and prostate cancer.

**Implications for Practice**

Although FDA-approved COVID-19 vaccines have been reported to be effective in patients with genitourinary cancers, there are still questions regarding the long-term immune response. This article evaluates the long-term response in this population up to 12 months after initial vaccination. This study provided important and novel insight into the long-term efficacy of COVID-19 vaccination among patients diagnosed with genitourinary cancers. The present data support continued efforts to provide immunization to patients with genitourinary cancers despite the concurrent use of standard of care systemic therapy and emphasizes the importance of strategies to preserve a robust immune response in this population.

## Introduction

As of November of 2022, over 630 million confirmed cases of severe acute respiratory syndrome-coronavirus-2 (SARS-CoV-2) infection have been reported worldwide, with at least <1% of these resulting in fatalities.^1^ The advent of coronavirus disease-19 (COVID-19) vaccines has had a transformative impact in reducing morbidity and mortality from the disease. At present, the US Food & Drug Administration (FDA) has issued emergency use authorizations for 3 vaccines—2 are RNA based (mRNA-1273 and BNT162b2), while a 3rd uses a more conventional adenoviral vector (Ad26.COV2.S).^2-4^ At the time of this report, nearly 5 billion individuals have been fully vaccinated worldwide.^1^

Early in the pandemic, several patient characteristics were noted to be associated with the development of severe COVID-19 and a worse prognosis.^5-10^ These included, but were not limited to, advanced age, obesity, and active or past history of malignancy. However, despite the increased susceptibility of patients with cancer, questions were raised regarding the efficacy and safety of COVID-19 vaccines in this patient population given the seminal studies leading to their FDA approval did not include such patients.^2-4^ In the last 2 years, a number of studies assessing the biological effects of COVID-19 vaccination in patients with active malignancies have been carried out across the world and there is now evidence supporting the general efficacy and safety of these interventions.^11-21^ Despite the proven benefits of commercially available COVID-19 vaccines, they have also raised new concerns. For example, it has been reported that clinical characteristics such as cancer type (eg, hematological versus solid malignancies) or type of treatment rendered could be associated with differences in the immune response mounted after vaccination.^13,22-24^

Furthermore, an important caveat across most published studies is the small number of patients with genitourinary malignancies included, making it challenging to make far reaching conclusions from these small numbers. This is especially important given the many different treatment approaches used for these cancer types that could impact the efficacy of COVID-19 vaccination. In addition, there is still a paucity of evidence regarding the long-term immunological response elicited by COVID-19 vaccines in this oncologic population. The present study sought to address this knowledge gap and assess the long-term effect of commercially available COVID-19 vaccination in patients with renal cell carcinoma (RCC), urothelial carcinoma, and prostate cancer. We hypothesize that patients with genitourinary malignancies regardless of the treatment or histology will achieve seroconversion at the 2-month type point.

## Materials and Methods

### Eligibility

Eligible patients were age 18 or over with a diagnosis of RCC, urothelial carcinoma, or prostate cancer. Patients could not have received prior COVID-19 vaccination, but ideally had an intent to receive any of the vaccines approved by the US FDA via emergency use authorization. Although a subsequent amendment to the protocol allowed for accrual of patients with lung cancer, this cohort accrued poorly and is not included in the current report. The study was approved by the City of Hope Scientific Review Committee and Institutional Review Board. All enrolled patients provided written informed consent prior to study entry, and all procedures related to the study were in accordance with the Declaration of Helsinki.

### Specimen Collection, Processing, and Analysis

Blood was collected at baseline (prior to COVID-19 vaccination). Once a dose of the vaccine was administered, blood was collected at 2, 6, and 12 months. Samples were collected in a serum-separating tube and were centrifuged within 1 h of collection; serum was transferred and stored in 2 mL screw-top tubes at −80 °C. Batched serum specimens were then assessed for antibody titers using the SCoV-2 Detect IgG ELISA Assay. Specimens and kit-provided controls were diluted (1:100) and added to antigen-coated wells and incubated at 37 °C for 1 h. After the incubation the plate was washed 6× in an automatic plate washer, and secondary antibodies conjugated to horseradish peroxidase (HPR) were added to the wells and incubated at 37 °C for 30 min. After another 6× washing, tetramethylbenzidine substrate was added and incubated in the dark at room temperature for 20 minutes, followed by a stop solution. Absorbance was measured at 450 nm. An immune status ratio (ISR) value was computed using the optical density of the study sample versus the optical density of specimens derived from unvaccinated healthy volunteers.

For T-cell receptor (TCR) sequencing, blood was collected using PAXgene tubes and stored at −80 °C. RNA was obtained from peripheral blood samples using PAXgene Blood miRNA Kit (PreAnalytiX) and stored at −80 °C. Next, iRepertoire Immune Repertoire Library Bulk reagent kits with i-R TCR Reagent System barcodes were used to convert the total RNA to cDNA and then independently amplify alpha and a beta (β) chain sequences. The resulting libraries were pooled equimolarly and sequenced using MiSeq v2 chemistry.

### Statistical Analysis

The primary objective of the study was to compare SARS-CoV-2 geometric mean ISR values at baseline and 2 months after one dose of vaccination. We expected to enroll a total of 135 patients to generate at least 90 evaluable subjects for the study. This would account for roughly 45% attrition by the 2-month timepoint for patients who decided not to get vaccinated after consent, as well as those unable to attend an appointment within the timeframe assigned for the 2-month blood collection. With 90 evaluable patients, we would have an 80% power to detect an effect size of 0.3 in the ISR GMT at month 2 in patients with genitourinary cancers (2-sided alpha = 0.05). A paired *t*-test was used to assess differences between ISR values at the previously specified collection timepoints. In addition, a non-paired *t*-test was used to compare the changes in ISR values of patients who received a booster to those who did not, and an ANOVA test was performed to compare the ISR values of patients based on cancer types and treatments rendered. For characterization of seroconversion, ISR values of<0.9 were considered seronegative and ISR values of ≥1.1 were considered seropositive. Intermediate values (ISR values between 0.9 and 1.09) were considered indeterminate and were retested.

### TCR Sequence Analysis

Raw bcl files were converted to a pooled fastq containing all samples from each sequencing experiment for R1 and R2, and sequence reads were assigned to individual samples using demultiplex (). Sequence reads for each sample were aligned to germline segments, and clonotypes were assembled and exported using mixcr (MiLaboratory, version 3.0.13), to generate a clonotype list of TCR-β chains in which each entry is characterized by a unique combination of V and J segments and the CDR3 nucleotide sequence. Samples with fewer than 100 000 sequence reads or 5000 clonotypes were excluded from downstream analysis, resulting in a total of 32 ­baseline:2-month sample pairs for statistical analyses. A 2-sided Fisher’s Exact test was performed for the counts of each clonotype (2-month sample versus the paired baseline control) to evaluate clonotype over-representation in either 2-month sample or baseline control. Clonotype counts for each sample were then normalized by taking the ratio of a clonotype to the sum of clonotypes in a sample. A 10-fold enrichment threshold over the patient’s paired sample and a fisher’s test *P*-value < 1e−25 was applied to determine time-variant enriched clonotypes. A database of all SARS-CoV-2 clonotypes was downloaded from VDJdb () on March 30, 2022 and filtered for CDR3 β chains with associated epitopes containing more than 10 entries. Time-variant enriched clonotypes were annotated using an exact match across CDR3 amino acid sequence. Statistical analysis and clonotype annotation were conducted in R statistical software (version 4.2).

## Results

### Patient Characteristics

**Figure 1.**
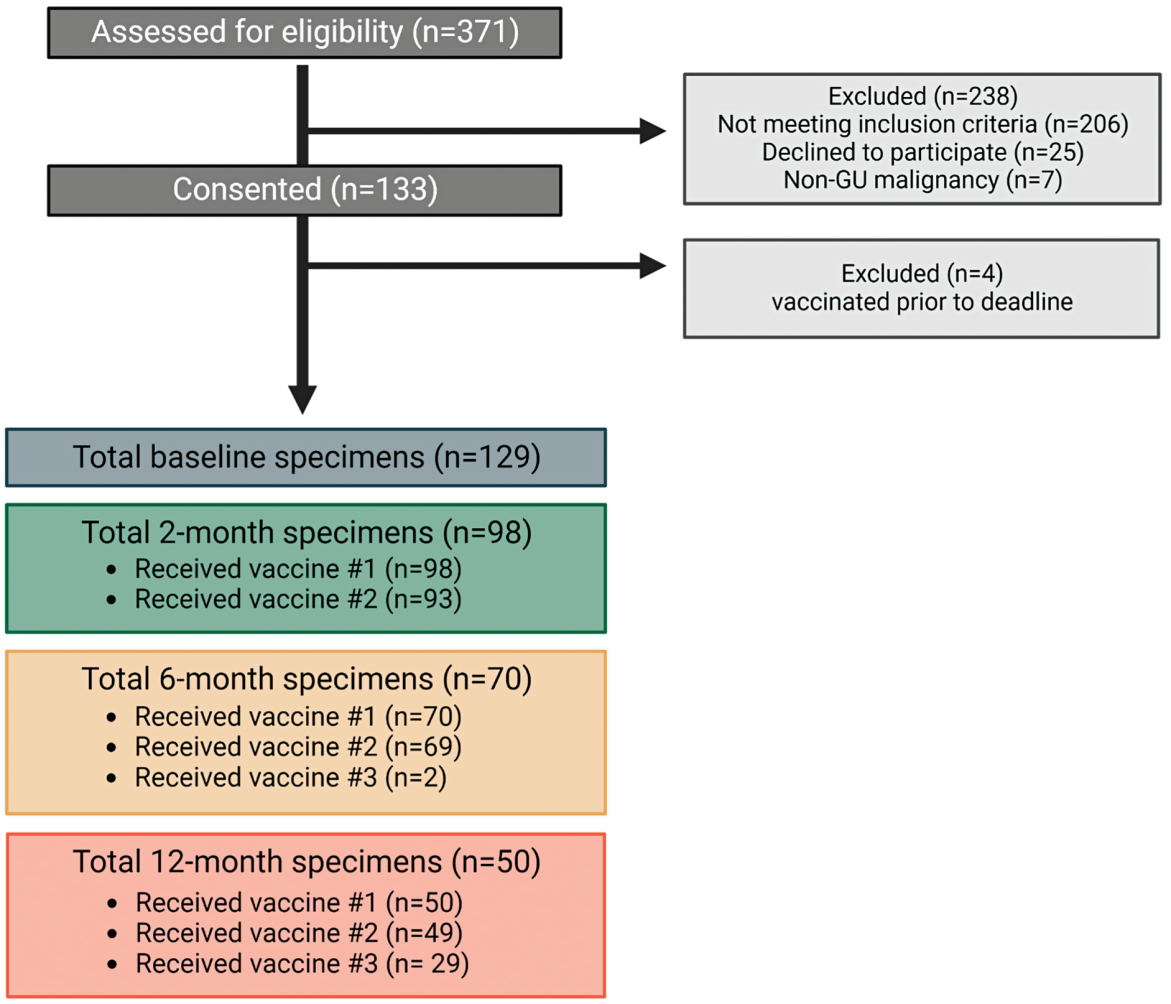
CONSORT diagram. Created with BioRender.com

In total, 133 patients with genitourinary malignancies were enrolled in our study. Of these, 129 patients received baseline blood draws, 98 received blood draws at 2 months (therefore evaluable for the primary endpoint of the study; see Fig. 1), with further blood samples obtained at 6 and 12 months obtained from 70 and 50 patients, respectively. A total of 54 (55.1%) patients with prostate cancer, 41 (41.8%) with RCC, and 3 (3.1%) with urothelial carcinoma were included; 82 (83.7%) were male, the majority (76.5%) were White, and the median age was 67 (IQR, 62-75; Table 1). Among evaluable patients, 87 (86.2%) were on systemic therapy. The most common systemic therapy regimens among this group were endocrine therapy (43.9%) and immune checkpoint inhibitors (ICIs; 26.5%), with the most common comorbidities being cardiovascular (46.9%) and endocrine/metabolic (39.8%).

**Table 1.** Patient characteristics (*n* = 98).

Characteristic	Value
Median age, years (IQR)	67 (62-75)
Gender—no (%)	
Male	82 (83.7)
Female	16 (16.3)
Race—no (%)	
White	75 (76.5)
Black	10 (10.2)
Asian	7 (7.1)
Unknown	6 (6.1)
Cancer type—no (%)	
Prostate cancer	54 (55.1)
Renal cell carcinoma	41 (41.8)
Urothelial carcinoma	3 (3.1)
Treatment type—no (%)	
Endocrine therapy	43 (43.9)
Immunotherapy	26 (26.5)
Targeted therapy	15 (15.3)
Chemotherapy	1 (1.0)
None	11 (11.2)
Vaccination type—no (%)	
BNT162b2 (Pfizer-BioNTech)	61 (62.2)
m-RNA-1273 (Moderna)	34 (34.7)
Ad26.COV2.S (Johnson & Johnson–Janssen)	3 (3.1)
Comorbidities—no (%)	
Cardiovascular	46 (46.9)
Endocrine and metabolic	39 (39.8)
Digestive	15 (15.3)
Pulmonary	11 (11.2)

### SARS-CoV-2 Antibody Response

#### Two Months After Vaccination

**Figure 2.**
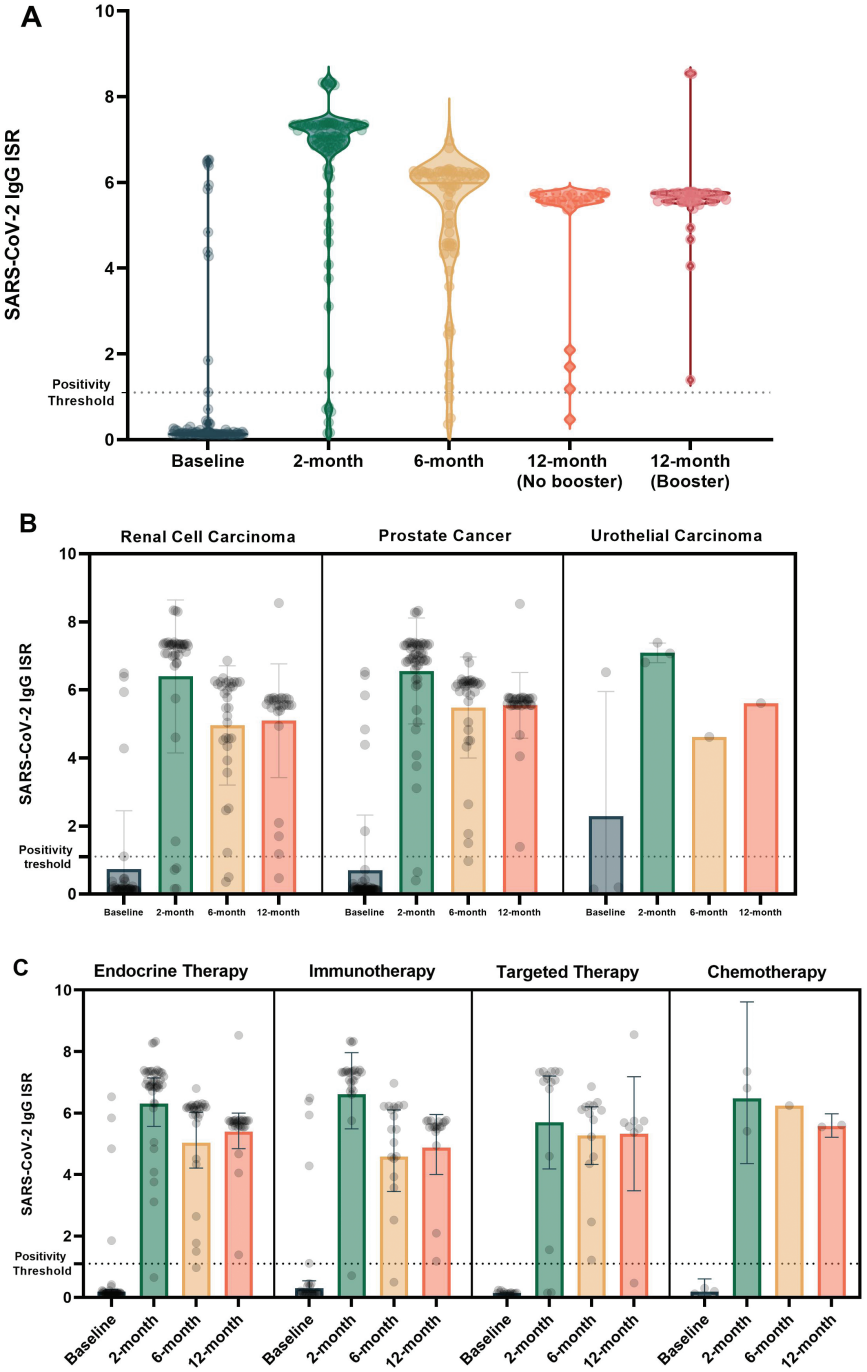
Change in geometric mean ISR values throughout the time in all evaluable patients (**A**), and in subsets based on histology (**B**) and treatment type (**C**).

At the 2-month timepoint, 98 evaluable patients were identified. Out of these in addition to their first dose, 94.9% also received second dose of the vaccination. The geometric mean ISR at baseline was 0.24 (95% CI, 0.19-0.31) and 5.59 at 2 months (95% CI, 4.76-6.55; *P* < .001; Fig. 2A). A total of 12 patients (12.2%) were noted to be seropositive at the time of study entry. All these patients had an increase in ISR values with vaccination; from 4.53 at baseline (95% CI, 3.15-6.49) to 7.36 (95% CI, 6.91–7.86) at 2 months (*P* = .004). Subtracting these patients from the analysis, the change in geometric mean ISR values remained significantly different, with an increase from 0.15 (95% CI, 0.14-0.17) at baseline to 5.49 (95% CI, 4.64-6.49) at 2 months (*P* < .001).

There were no significant differences in the change of ISR based on cancer type or treatment rendered (*P* = .72 and *P* = .22, respectively; Figs. 2B–2C). A total of 6 patients (6.1%) did not achieve seroconversion based on the aforementioned definitions. These patients had a mean ISR of 0.16 (95% CI, 0.12-0.21) at baseline, and a mean ISR of 0.39 (95% CI, 0.18-0.85) at 2 months (*P* = .03). Detailed characteristics of these patients are provided in Supplementary Table S1.

#### Six Months After Vaccination

At the 6-month timepoint, paired samples from 70 patients were obtained. In this cohort, 69 (98.6%) patients had their second dose of the vaccine and 2 (3%) received the third dose. Geometric mean ISR was 4.66 (95% CI, 4.04-5.38). When compared to the 2-month timepoint, a significant decrease in ISR values was seen (*P* < .0001; Fig. 2A). However, as seen at the 2-month timepoint, there were no significant differences in the mean ISR values at 6 months based on cancer type, or treatment rendered (*P* = .76 and *P* = .15, respectively). Notably, only 1 out of the 6 seronegative patients (16.7%) achieved seroconversion at the 6-month timepoint despite being fully vaccinated.

#### Twelve Months After Vaccination

At the 12-month timepoint, paired samples were obtained for 50 patients. Of these, 29 (58%) had received a booster and had a geometric mean ISR of 5.47 (95% CI, 4.90-6.10) while 21 patients (42%) had not received one and had a geometric mean ISR of 4.19 (95% CI, 3.08-5.70; *P* = .05). When compared to the ISR values obtained at the 6-month timepoint, patients receiving a booster dose had an absolute increase of 1.55 compared to those who did not receive a booster dose (*P* = .04).

Akin to what was observed at 6 months, there were no significant differences in mean ISR values were observed based on cancer type or treatment rendered. Additionally, samples from 3 of 6 patients who failed to seroconvert after the second dose of the vaccine were obtained. Notably, all of these patients had seroconverted by the time of the 12-month blood draw.

### TCR Sequencing

A total of 4 321 419 clonotypes were identified in the 32 baseline:2-month sample pairs with a per-sample median of 269 119 clonotypes (IQR: 187 035). Though a significant cohort-wide clonotype signal was not apparent in these data, there were some individual-level trends identified. Seven patients were found to have time-variant spike-specific clonotypes in their 2-month sample. Patient 20734-015 and patient 20734-119 had 3 and 2 unique time-variant spike-specific clonotypes, respectively, and the remaining 5 patients all had one spike-specific clonotype identified in the 2-month samples. In total, there were 10 spike-specific TCR clonotypes identified in the 7 patients with 8 of those being unique CDR3aa sequences. There were 5 unique V genes and 3 unique J genes across the 10 clonotypes. The most common spike-epitope was YLQPRTFLL (8/10 clonotypes), followed by one each for epitopes HWFVTQRNFYEPQII and QYIKWPWYI.

## Discussion

To our knowledge, our data represent the largest study reporting a 12-month follow-up after COVID-19 vaccination in patients with genitourinary cancers to date. In our series, over 93% of patients achieved seroconversion 2 months after receiving US FDA-approved vaccines. Initial studies in solid tumors focused on seroconversion at earlier timepoints (eg, within 21 days of initiation of vaccination) and cited inadequate rates of seroconversion.^12^ With our uniform collection of specimens at 2 months, the rates of seroconversion appear significantly higher and are consistent with more recent series reporting adequate immune responses after vaccination in patients with solid tumors.^11,13,15-19,25,26^

Notably, our analysis of antibody titers at the 6-month timepoint showed evidence of significantly decreased ISR values from the 2-month timepoint, suggesting waning humoral immunity. This finding mirrors prior studies that also reported this phenomenon and highlighted the importance of administering a booster dose after completion of an initial vaccination series.^20,27,28^ This strategy is further supported by the results of our 12-month follow-up which demonstrated a significant increase in ISR values between patients receiving a booster dose compared to those who did not.

We believe it is critical to evaluate seroconversion following COVID-19 vaccination in a disease-specific context. Among studies that have been reported to date for patients with solid tumors, patients with genitourinary cancers constituted only 8%-17% of the sample assessed.^11-13,29^ In the current study of primarily patients with prostate and RCC, the treatments rendered were representative of the respective disease types—patients with prostate cancer most frequently received endocrine therapy, while patients with RCC most frequently received either targeted therapy or immunotherapy. Prostate cancer has been purported to have a complex interplay with SARS-CoV-2, with expression of the angiotensin-converting enzyme (ACE-2) receptor in prostate epithelial cells.^30^ Entry of SARS-CoV-2 into the cell depends on the binding of viral proteins to ACE-2 and the androgen-regulated gene TMPRSS2.^31^ Use of endocrine therapy for prostate cancer has been suggested to have a protective effect by modulating these proteins.^32^ Although the mechanistic impact of endocrine therapy on immunogenicity is not well documented, smaller series supports our observation that most patients with prostate cancer receiving endocrine therapy achieve seroconversion following COVID-19 vaccination.^13^

Vascular endothelial growth factor (VEGF)-directed therapies for RCC can modulate immunity by increasing MHC class I expression, antigen processing/presentation, T-cell activation, and programmed death-ligand 1 (PD-L1) expression.^33,34^ In a previous study with influenza vaccination, patients receiving sunitinib or sorafenib developed a similar seroprotection compared to healthy volunteers.^35^ The link between ICIs and immunogenicity is perhaps more implicit and stems from their mechanism of action, and thus one could anticipate enhanced and sustained antibody titers in the context of vaccination. Indeed, this association has been borne out in studies exploring the influenza vaccine in patients receiving ICIs.^36,37^ In our study, in line with previous hypotheses, no significant differences among patients receiving endocrine therapy, targeted therapy, or immunotherapy were seen across any of the prespecified timepoints, indicating that the concurrent use of these therapies does not represent a barrier to obtaining a robust immune response to the COVID-19 vaccination among patients with genitourinary malignancies. These findings complement previously reported data from Grivas et al, suggesting that these therapies do not adversely affect the severity of infection among patients with cancer.^9^

As suggested by our results, the cellular immune response across evaluable patients from our study population is largely dominated by distinct TCR sequences against just a few epitopes of SARS-CoV-2. This is possibly due to TCR bias elicited by vaccination.^38^ Similarly, the most common spike-epitope identified in our cohort, YLQPRTFLL, has been characterized as an immunodominant epitope and has previously been described in multiple studies to be the target of strong CD8 + T-cell responses in infected HLA-A*02:01 + individuals.^39-43^ Approximately 30% of patients who exhibited spike-specific clonotypes at the 2-month timepoint displayed unique specific TCR clonotypes in response to vaccination. This is likely explained by the influence of interindividual variability arising from diverging genetic and epigenetic factors.^38^

Limitations of our study include the fact that this represents a single-center experience. However, this allowed us to maintain a more rigorous schedule of sample collection with baseline blood assessment. Monitoring of antibody titers was frequent across each cycle of therapy and extended to 6- and 12-month blood draws. However, due to our strict timeframe for collection patient attrition was observed at the later time points as the lab appointments fell outside of our prespecified criteria. Another limitation of our study is the rather limited sample size, especially regarding the enrollment of patients diagnosed with urothelial carcinoma—with only 3 patients in this subset, we can draw limited inferences regarding the efficacy of vaccination in this population. Similarly, few patients in our study received chemotherapy, a modality frequently used in the treatment of urothelial and prostate cancer, and thus our results may be less applicable in this context as well. Finally, our study did not account for potentially confounding factors such as age, gender, ethnicity, or medical comorbidities.

Despite these shortcomings, our study provides important and novel insight into the long-term efficacy of COVID-19 vaccination among patients diagnosed with genitourinary cancers. Patients with advanced prostate cancer receiving endocrine therapy and patients with RCC receiving targeted therapy and immunotherapy were well represented within this cohort. Our data support continued efforts to provide immunization to patients with genitourinary cancers despite the concurrent use of standard of care systemic therapy and further emphasizes the importance of strategies to preserve a robust immune response in this vulnerable population.

## Supplementary Material

oyad067_suppl_Supplementary_Table_S1

## Data Availability

The data underlying this article will be shared on reasonable request to the corresponding author.
