# Supplementary material for: Twelve-Month Follow-up of the Immune Response After COVID-19 Vaccination in Patients with Genitourinary Cancers: A Prospective Cohort Analysis
Source: Oncologist. 2023 Mar 27;28(9):e748–55. doi: 10.1093/oncolo/oyad067 (PMC10485287; doi:10.1093/oncolo/oyad067)
Supplement: oyad067_suppl_Supplementary_Table_S1 [file oyad067_suppl_supplementary_table_s1.docx]

**Supplemental Table 1.** Detailed characteristics of patients who did not achieve seroconversion at month 2 following COVID-19 vaccination.

|  | **Patient 1(BS)** | **Patient 2 (RL)** | **Patient 3 (GJ)** | **Patient 4 (MH)** | **Patient 5 (JL)** | **Patient 6 (MC)** |
| --- | --- | --- | --- | --- | --- | --- |
| **Sex** | Male | Male | Male | Male | Male | Female |
| **Age** | 64 | 73 | 82 | 63 | 59 | 57 |
| **Race** | White | White | White | White | White | White |
| **BMI** | 19.6 | 24.7 | 42.2 | 25.9 | 39.9 | 23.6 |
| **Vaccination date** | 3/12/2021 | 2/4/2021 | 2/8/2021 | 3/14/2021 | 3/18/2021 | 8/10/2021 |
| **Vaccine type** | Pfizer | Moderna | Pfizer | Johnson & Johnson | Pfizer | Pfizer |
| **ISR baseline** | 0.12 | 0.26 | 0.14 | 0.13 | 0.15 | 0.18 |
| **ISR month 2** | 0.16 | 0.76 | 0.4 | 0.65 | 0.71 | 0.15 |
| **ISR month 6** | 1.22 | 0.36 | - | 0.36 | 0.5 | - |
| **Received booster** | Yes | No | No | No | Yes | - |
| **ISR month 12** | 5.69 | 1.7 | - | - | 5.74 | - |
| **Allergies** | Kiwi extract | Erythromycin, malarone, ciprofloxacin, propofol | Hydrocodone-acetaminophen Acyclovir | NKA | NKA | NKA |
| **Cancer type** | RCC | RCC | Prostate cancer | Prostate cancer | RCC | RCC and Lymphoma |
| **Metastatic disease** | Yes | Yes | No | No | Yes | Yes |
| **Metastatic sites** | Bone, lung, heart, retroperitoneal | Muscle, lung, adrenal | N/A | N/A | Bone | Lung, adrenal, bone |
| **Treatment** | Cabozantinib + nivolumab | Not on active treatment | Not on active treatment | Leuprolide | Nivolumab | Experimental immunotherapy |
| **Comorbidities** | Hypertension | Rheumatoid arthritis, vasculitis | Chronic hypotension, hyperlipidemia | Basal cell carcinoma, melanoma | Chronic kidney disease | Diabetes |
| **Immune modulator drugs** | None | Methotrexate Methylprednisolone | Fludrocortisone | Prednisone | None | Rituximab |

**Abbreviations:** BMI, body-mass index; ISR, immune status ratio; RCC, renal cell carcinoma; NKA, no known allergy; N/A, not applicable.
